# Lithium-Ion Glass Gating of HgTe Nanocrystal Film with Designed Light-Matter Coupling

**DOI:** 10.3390/ma16062335

**Published:** 2023-03-14

**Authors:** Stefano Pierini, Claire Abadie, Tung Huu Dang, Adrien Khalili, Huichen Zhang, Mariarosa Cavallo, Yoann Prado, Bruno Gallas, Sandrine Ithurria, Sébastien Sauvage, Jean Francois Dayen, Grégory Vincent, Emmanuel Lhuillier

**Affiliations:** 1 Sorbonne Université, CNRS, Institut des NanoSciences de Paris, INSP, 75005 Paris, France; 2 ONERA-The French Aerospace Lab, 6 Chemin de la Vauve aux Granges, 91123 Palaiseau, France; 3 Laboratoire de Physique et d’Etude des Matériaux, ESPCI-Paris, PSL Research University, Sorbonne Université, CNRS, 10 Rue Vauquelin, 75005 Paris, France; 4 CNRS, Centre de Nanosciences et de Nanotechnologies, Université Paris-Saclay, 91120 Palaiseau, France; 5 IPCMS-CNRS, Université de Strasbourg, 23 Rue du Loess, 67034 Strasbourg, France; 6 Institut Universitaire de France, 1 Rue Descartes, CEDEX 05, 75231 Paris, France

**Keywords:** field effect transistor, solid electrolyte, HgTe, nanocrystals, nanophotonics, light resonator, infrared detection

## Abstract

Nanocrystals’ (NCs) band gap can be easily tuned over the infrared range, making them appealing for the design of cost-effective sensors. Though their growth has reached a high level of maturity, their doping remains a poorly controlled parameter, raising the need for post-synthesis tuning strategies. As a result, phototransistor device geometry offers an interesting alternative to photoconductors, allowing carrier density control. Phototransistors based on NCs that target integrated infrared sensing have to (i) be compatible with low-temperature operation, (ii) avoid liquid handling, and (iii) enable large carrier density tuning. These constraints drive the search for innovative gate technologies beyond traditional dielectric or conventional liquid and ion gel electrolytes. Here, we explore lithium-ion glass gating and apply it to channels made of HgTe narrow band gap NCs. We demonstrate that this all-solid gate strategy is compatible with large capacitance up to 2 µF·cm^−2^ and can be operated over a broad range of temperatures (130–300 K). Finally, we tackle an issue often faced by NC-based phototransistors:their low absorption; from a metallic grating structure, we combined two resonances and achieved high responsivity (10 A·W^−1^ or an external quantum efficiency of 500%) over a broadband spectral range.

## 1. Introduction

As colloidal growth of nanoparticles has gained maturity, nanocrystals (NCs) have become viable building blocks for optoelectronics. Using narrow band gap materials, it is possible to address the infrared (IR) part of the electromagnetic spectrum [1,2]. At such wavelengths, NCs offer an interesting alternative to epitaxially grown semiconductors to design cost-effective devices [3]. This has led to the demonstration of efficient light-emitting devices [4,5,6,7] as well as effective light sensors [8,9,10], including focal plane arrays used to image near- and mid-IR light [11,12].

Though a high degree of control is achieved for the growth of this material, its doping remains [13] a challenge, and the material may not behave as an intrinsic semiconductor. This affects the final device performance. Thus, it is of utmost interest to couple NC films with a gating method to enable an agile tunability of the carrier density. As a result, the phototransistor appears to be a remarkable evolution of the photoconductive geometry, since the gate bias can be used as a knob to control the magnitude of the dark current. Phototransistors are built around a field-effect transistor [14] (FET), in which, in addition to drain/source electrodes and a channel based on a NC film, the gate appears as a central building block. The gate is used as a capacitor and, under applied bias, it generates charges, tuning the carrier density in the FET channel.

Various gate technologies have been coupled to NC films [15]. The most conventional method continues to rely on dielectrics such as SiO_2_ [9,16,17]. Since this material is commercially available and technologically mature, it presents the clear benefit of being easy to implement. Moreover, the fast gate bias sweep and the ability to operate at low temperatures are other key advantages of this technology. The low dielectric constant of silica, on the other hand, requires a large bias operation, possibly close to the material breakdown. High-k materials such as alumina, ZrO_x_ [18,19] or HfO_2_ are the natural alternatives [20,21,22,23]. They present the advantage of an all-solid strategy similar to silica but require an additional fabrication step with the deposition of an insulating layer. Despite a limited number of examples [24,25], ferroelectric gating coupled with a NC film offers a strategy to achieve even higher capacitance FETs. The main idea behind ferroelectric gating is to take advantage of the divergence of a ferroelectric material’s dielectric constant close to its Curie temperature. However, this limitation tends to reduce the operating temperature ranges. When higher gate capacitances are required, gating methods based on ion displacement become the most effective. Contrary to the previous methods, electrolytes allow for bulk gating. This ability to gate a thick film is critical for the design of phototransistors, which require a film thickness suitable to the light absorption depth [26,27]. Electrolytes are already commonly used to control the carrier density of a NC population [28,29,30]. The usual method is to introduce a liquid or ion gel electrolyte, which may bring additional constraints related to liquid handling and moisture sensitivity; this is why a solid electrolyte appears easier to implement. The use of ionic glasses has been a step in this direction; LaF_3_ glass presents, for example, mobile fluoride vacancies which can be used to induce gating [31,32,33].

When applied to infrared phototransistors, the gate has to fulfill some specific constraints, such as being:

1. Compatible with operations below room temperature;
2. Solid state-based to be conveniently integrated into a device;
3. Associated with a large capacitance to tune the relatively large thermally activated carrier density observed in narrow band gap NCs.

In this perspective, new materials compatible with this series of constraints must be identified. For a long time, the battery field was using solid electrolytes, and their potential for field-effect transistors has been investigated for 2D materials [34,35,36,37] and superconductor-based [38] FETs, but they remain unused for NC-based FETs. Here, we explore the potential of this strategy to design a FET based on HgTe NCs [39], presenting an absorption in the extended short-wave infrared. We show in particular that this lithium-ion glass substrate tends to promote hole injection over electron injection compared to what is observed with a silica gate. In the last part of the paper, we propose an update of the design to engineer light-matter coupling in this system and achieve high absorption from a thin NC film. This strategy allows a large responsivity (R > 10 A·W^−1^), three orders of magnitude larger than the value obtained without a light management strategy.

## 2. Materials and Methods

### 2.1. Nanocrystal Growth

**Chemicals**: Mercury chloride (HgCl_2_; Sigma-Aldrich, St. Louis, MO, USA, 99%), **Mercury compounds are highly toxic. Handle them with special care.** Tellurium powder (Te; Sigma-Aldrich, 99.99%), trioctylphosphine (TOP; Alfa Aesar, Haverhill, MA, USA, 90%), oleylamine (OLA; Thermo Fisher Scientific, Illkirch, France, 80–90%), dodecanethiol (DDT; Sigma-Aldrich, 98%), ethanol absolute anhydrous (VWR, Radnor, PA, USA), methanol (VWR, >98%), isopropanol (IPA; VWR), hexane (VWR, 99%), 2-mercaptoethanol (MPOH; Merck, Rahway, NJ, USA, >99%), N,N dimethylformamide (DMF; VWR), toluene (VWR, 99.8%), acetone (VWR), methylisobutylketone (MIBK; VWR, >98.5%)

All chemicals were used without further purification except oleylamine, that was centrifuged for 5 min at 6000 rpm before use.

**1 M TOP:Te precursor**: The procedure was taken from ref [40].

**HgTe NCs synthesis with band-edge at 4000 cm^−1^**: The procedure was inspired by ref [41].

**HgTe ink**: The procedure was inspired by ref [42]. Before ink deposition, the substrate was placed in an oxygen plasma cleaner for 2 min to promote adhesion. The ink was spin-coated on the substrate at 4000 rpm (with $2000\;\mathrm{rpm}\cdot s^{-1}$ acceleration) for 1 min. This gave a homogeneous film between 100 nm and 250 nm, as can be seen in the height profile obtained from the profilometer, shown in Figure S1 (Supplementary Materials). The speed of spin coating and ink concentration were used to tune the film thickness.

### 2.2. Material Characterization

**Infrared spectroscopy**: We conducted measurements in attenuated total reflection (ATR) mode using a Fischer Nicolet iS50. The spectra were acquired with a 4 cm^−1^ resolution and averaged 32 times. The photocurrent spectra were obtained while biasing the sample with a Femto DLPCA 200 current amplifier; the signal was then magnified by the same amplifier and the output signal was fed into the spectrometer. For polarized measurements, a polarizer aligned with the electrode digits (or perpendicular to them) was added on the optical path. The background was then acquired in the presence of the polarizer in both polarizations.

**Transmission electron microscopy**: NC solution was drop-casted onto a TEM grid and dried on a paper. The grid was degassed overnight to suppress solvent and volatile species.

### 2.3. Electrical Characterization

**Electrode fabrication**: The solid electrolyte was purchased from MTI (ref EQ-CGCS-LD). It is a ceramic based on Li_2_O-Al_2_O_3_-SiO_2_-P_2_O_5_-TiO_2_. The substrate was cleaned with acetone and rinsed with ethanol and isopropanol, before being dried by a nitrogen jet gas. First, LOR 3A, a sacrificial release layer, was spin-coated and baked at 160 °C for 180 s. Then, AZ 1505 resist was spin-coated and annealed at 105 °C for 90 s. Laser lithography (Heidelberg µPG 101) was used to conduct the lithography procedure. The resist was developed for 20 s in AZ 726 developer, and finally rinsed in water. Then, 3 nm of titanium and 47 nm of gold were evaporated using an e-beam evaporator (Plassys MEB 550s). For lift-off, the substrate was dipped in a PG remover bath for 1 h, and finally rinsed using DI water. The substrate was then dried and electrically tested to check that there was no electrical short. The electrodes were interdigitated electrodes, as schematized in Figure S2, with 25 pairs of digits (i.e., 49 spacings). Each digit was 700 µm long and spaced from its neighbor by 10 µm.

**Electrical measurements:** The drain and source contacts of the sample were cleaned using a cotton swab moistened with acetone. The sample was glued using a silver paste on a substrate of silicon covered with a gold layer and mounted on the cold finger of a cryostat. The gold layer was used as a gate. The transfer curves and the IV curves were measured using a Keithley 2634B SourceMeter; this device controls both the gate-source and the drain-source voltages, measuring the relative currents.

**Photocurrent measurements:** The time response measurements and the responsivity measurements were performed using the scope mode of a MLFI Lock-In Amplifier (Zurich Instruments, Zürich, Switzerland). In this case, both the drain-source and the gate-source voltages were controlled by the Lock-In Amplifier. Figure S4 schematized the setup used for the responsivity measurements. A wavefunction generator was used to trigger the laser emission and the oscilloscope; the current was converted into voltage by the Femto DLPCA-200 amplifier and fed into the oscilloscope (Tektronix MDO 3102). From the time trace we can extract both the photocurrent amplitude and the time response.

**Impedance Measurements:** The MLFI Lock-In Amplifier (Zurich Instruments, Zürich, Switzerland) was used to perform impedance measurements in the range from 0.1 Hz to 500 kHz. We used an AC voltage of 100 mV. No DC voltage was applied, exploring the temperature dependence of the impedance. We first deposited two gold layers on both sides of a Li-based substrate. The surface of the deposited gold layer was determined a posteriori using a camera and digital processing of the image. The area of the device was 8.9 mm^2^. The device was glued on a substrate of silicon coated with a gold layer using silver paste. The sample was then connected inside the cryostat, and the top and the back sides were connected to the Lock-In Amplifier.

**Noise measurement:** Current from the device (at 0.7 V bias, kept in the dark) was amplified by a Femto DLPCA-200, then fed into a SRS SR780 spectrum analyzer. The sample was mounted on the cold finger of a closed cycle cryostat.

**Detectivity determination:** The specific detectivity (in Jones) of the sample was determined using the formula: $D^{*}=\frac{R\sqrt{A}}{S_{I}}$, where *R* (in A·W^−1^) is the responsivity, S_I_ is the noise (A/$\sqrt{Hz}$) and A is the area of the device (cm²).

### 2.4. Optical Resonator

**Determination of the optical index**: We used spectrally resolved ellipsometry to determine the complex optical index of the different materials involved in the device and later conduct electromagnetic simulations.

**Spectroscopic ellipsometry**: The procedure was similar to the one used in ref [43]. The spectroscopic ellipsometry measured the change in the polarization state between the incident and the reflected light. This was done by measuring the angles ψ and Δ. The following relation applies:

(1) $$\rho =\frac{r_{p}}{r_{s}}=\left|\frac{r_{p}}{r_{s}}\right|e^{i\left(\delta_{p}-\delta_{s}\right)}=\tan\psi e^{i\Delta }$$

where *r_p_* and *r_s_* are the reflection coefficients of *p* and s polarized light, respectively, and where $\delta_{p}$ and $\delta_{p}$ are the phase shifts in reflection in *p* and s polarizations, respectively. The measurements were performed on a V-VASE ellipsometer (J.A. Woollam) in the 500–2000 nm range with steps of 10 nm and with angles of incidence of 50°, 60° and 70°. For the Li-based substrate, the real part is shown in Figure S6 and was later used as a free parameter to fit experimental data; we found that *n* = 1.8 gives a reasonable fit. To account for the diffusive character of the substrate, we used k = 1 for the substrate.

Complementarily, note that for the gold complex optical index, we used ref [44]. Figure S7 provides the imaginary part of the complex optical index of the HgTe NC film. The real part of the optical index was taken as constant and equal to 2.2 according to ref [43].

**Electromagnetic simulation**: Simulations were conducted using COMSOL 5.6, a software using the Finite Element Method. The array of resonators was modelled using the RF module in 2D geometry. Floquet periodic boundary conditions were used to describe the periodicity in one unit cell. On both sides (top and bottom), we defined perfectly matched layers (PML) to absorb all outgoing waves and prevent nonphysical reflections. The absorption came from emw. Qe, a value corresponding in COMSOL to the power density dissipated in W·m^−3^. In air, there is no diffracted order for wavelengths above the electrode period. For shorter wavelengths, the energy propagating in the diffracted orders is absorbed by PML. On top of the resonator inside air, a port condition was used to define the incident wave, either in TE or TM polarization. An automatic mesh was used in these simulations with a predefined “extremely fine” mesh, which means that the maximum element size was 140 nm, except for the PML, where a mapped mesh was used with a distribution of 12 elements.

To enhance the device absorption, we kept the same interdigitated device geometry and just updated some geometrical factors, as shown in Figures S8 and S12. We tuned the period, the size of the digit and the film thickness so that light absorption resonances were generated. Along the TE polarization, we designed a guided mode resonance. This mode is dispersive and split in two modes with angles as revealed by the dispersion map along the TE polarization (see Figure S9a). In TM mode, we observed one main resonance that we designed to be red shifted compared to the TE mode. This mode is nondispersive (see TM dispersion map in Figure S9b), and while it is not affected by the digit size (i.e., no shift of the resonance peak for various digit sizes, as shown in Figure S11), it can strongly be affected by the film thickness (i.e., a strong shift of the resonance peak away from the exciton peak, as shown in Figure S10), which suggests a vertical Fabry-Pérot resonance.

**Fabrication of the device with resonator** The fabrication of the optimized photoresponsive device was accomplished in two steps of lithography. First, the main contact pads were fabricated using optical lithography, and then the interdigitated pattern was fabricated using e-beam patterning.

**Electrodes for electrical contacts:** A lithium-ion glass substrate was initially rinsed with acetone and isopropanol to remove dust and organic contaminants. An additional O_2_ plasma cleaning step was performed for 5 min. The TI Prime adhesion promoter was spin-coated onto the substrate and baked on a hot plate at 120 °C for 2 min. Subsequently, the substrate was spin-coated with AZ5214E photoresist and baked at 110 °C for 1 min 30 s. The first UV exposure through a mask was 1 s, as the substrate was very strongly scattering light, and this was followed by the image reversal bake step at 125 °C for 2 min. A flood exposure for 40 s without the mask was then performed. The resist film was developed in AZ726 MIF with a development time of 30 s, followed by DI water rinse, N_2_ gun and finally an oxygen plasma over 5 min. The next step was to deposit 5 nm of Cr and 80 nm of Au on the substrate with a thermal evaporator. Afterwards, the substrate stayed for 2 h in acetone for lift-off. The obtained pattern is shown Figure S12a.

**Fabrication of gold nano-stripes:** The procedure was similar to the one used in ref [45]. We used a Raith eLine e-beam lithography system for the fabrication of the nano-stripes. First, a 400 nm thick PMMA layer was spin-coated onto the substrate patterned with macroscopic electrodes. The resist was then baked at 180 °C for 2 min. We deposited 10 nm of Al with a thermal evaporator in order to reduce the charging effect. The operating bias of the electron beam was set to 20 kV, and the aperture was set to 10 μm. The dose was set to 170 μC·cm^−2^. The resist was first dipped for a duration of 15 s in a KOH solution in order to remove the aluminum layer, and then it was developed in a solution of MIBK: isopropanol (1:3 in volume) for 45 s and rinsed with isopropanol. An oxygen plasma over 5 min was done to remove traces of resist. 5 nm Cr and 80 nm Au were then deposited with a thermal evaporator. The substrate stayed in hot acetone (45 °C) for 2 h after letting it overnight for lift-off. The obtained pattern is shown in Figure S12b–d.

For resonator measurements, a thicker film compared to transistor measurements was needed (>200 nm). Thicker films can be obtained mostly by updating the spin coating condition, which is conducted at reduced speed. The ink is spin-coated on the substrate at 1500 rpm (at 500 rpm·s^−1^ acceleration) for a duration of 5 min (after initial DMF deposition on the substrate with the same speed in order to help adhesion), followed by a fast spin coating step to dry the edges (3000 rpm for 2 min, with 500 rpm.s^−1^). This gives a homogeneous deposition around 225 nm; see Figure S1 for an image of the electrode after functionalization with the NC film.

## 3. Results and Discussion

### 3.1. Material of Interest with Short Wave Infrared Band Gap

**Figure 1:**
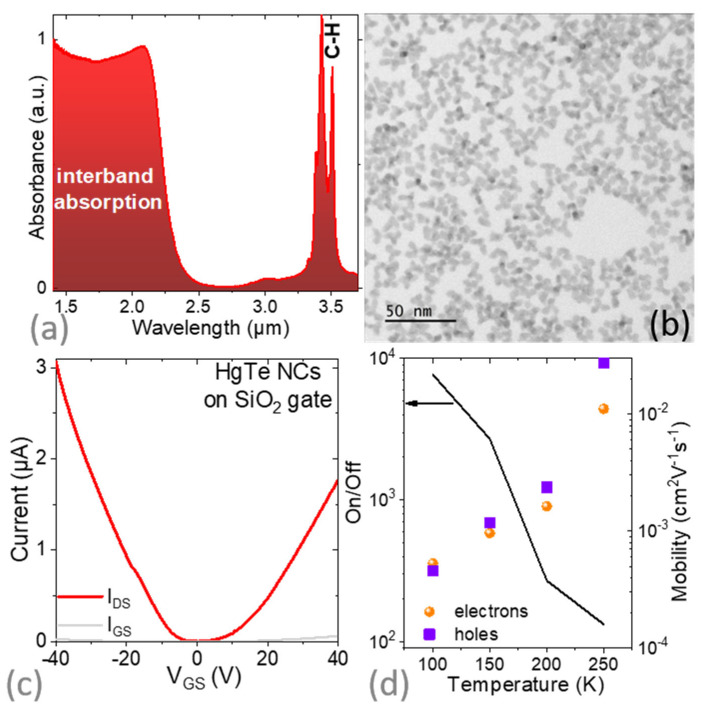
(**a**) Absorption spectrum of the HgTe NCs measured in attenuated total reflection mode. The doublet at around 3.4 µm is related to the resonance of the C-H bond. (**b**) Transmission electron microscopy image of the HgTe NCs. (**c**) Transfer curve (drain I_DS_ and gate current I_GS_ as a function of applied gate bias under the constant drain-source bias of 100 mV) measured at 150 K for a field-effect transistor whose channel is made of a HgTe NC film while the gate is SiO_2_ (300 nm SiO_2_ on top of highly doped Si). (**d**) On/off ratio (solid line) and carrier mobility (scatter) as a function of the temperature for a FET whose channel is made of HgTe NCs and the gate of 300 nm SiO_2_.

To design a short wave-infrared (SWIR) absorbing phototransistor, we proceeded in two informative steps. In the first step, we relied on a doped silica substrate. We used HgTe NCs as the material for the FET channel, since HgTe combines both a tunable absorption edge in the short- and mid-wave infrared together with stable photoconductive properties. The nanocrystals were obtained using the Keuleyan’s procedure [41]. HgTe NCs present a tripodic shape according to electronic microscopy (Figure 1b) and a band edge at 2 µm with a cut-off wavelength around 2.5 µm (see the absorption spectrum in Figure 1a). In addition to the electronic interband transition attributed to HgTe, the absorption spectrum depicts a doublet that is attributed to the C-H bond resonance at around 2900 cm^−1^. Several steps of cleaning were conducted in order to reduce the relative magnitude of the C-H resonance close to the one of the NC exciton. When a similar magnitude was obtained, thin conductive and photoconductive films of such NCs were obtained by preparing an ink [16,42]. In this case, the native insulating long ligands (dodecanethiol) were stripped off and replaced by a mixture of a short thiol (mercaptoethanol) and ions (HgCl_2_) dissolved in dimethylformamide (DMF). The obtained ink can be spin-coated to form a thin film. The film thickness can be tuned with the deposition conditions in the 100 to 300 nm range for the device under investigation in this study.

Once deposited on a doped Si/SiO_2_ substrate on which gold interdigitated electrodes have been fabricated, the material presented an ambipolar character. We observed both holes (under negative bias) and electrons (under positive gate bias), as shown in the transfer curve (see Figure 1c). The current modulation was around two orders of magnitude at 250 K and increased to almost four decades at 100 K (see Figure 1d). This increase in current modulation was mostly due to a reduction in the off current caused by the reduced carrier density activation when the temperature drops.

**Figure 2:**
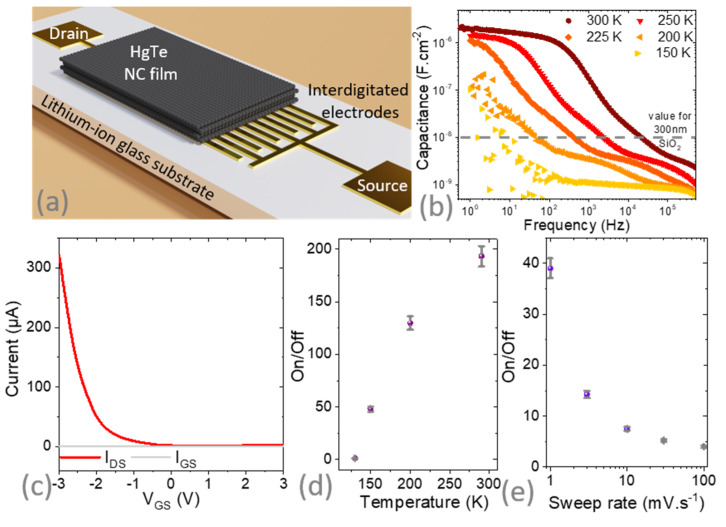
(**a**) Schematic of the FET whose channel is made of HgTe NC film and whose gate is made of a lithium-ion glass substrate. The substrate is used as a gate and the gate electrode is taken on the back side of the substrate. (**b**) Capacitance of the lithium-ion glass substrate as a function of the signal frequency for various temperatures. (**c**) Transfer curve for the FET depicted in part a at room temperature (V_DS_ = 500 mV). On/off ratio (i.e., ratio of the maximum current over the minimum current in the transfer curve) for the same FET as a function of temperature (**d**) and gate bias sweep rate at 200 K (**e**). Error bars in part d and e are determined by several repetitions of the transfer curve.

The carrier mobility can be determined from the transfer curve with:

(2) $$\text{µ}=\frac{L}{WC_{\Sigma }V_{DS}}\frac{\partial I_{DS}}{\partial V_{GS}}$$

where *L* is the electrode spacing (10 µm), *W* is the electrode length (49 × 2.5 mm), *C_Σ_* is the surface capacitance as measured in Figure 2b and *V_DS_* is the drain source bias. We noticed a clear thermal activation of the mobility (Figure 1d), which was consistent with the hopping conduction occurring in a disordered array of polydisperse NCs. Electron and hole mobilities were similar in magnitude and dropped by a factor of 50 as the temperature was reduced from 250 to 100 K.

### 3.2. Gating Using Solid Electrolyte

In the second step, we designed a FET using a lithium-ion glass substrate. The latter is commercially available from MTI and is a ceramic of oxide materials (Li_2_O-Al_2_O_3_-SiO_2_-P_2_O_5_-TiO_2_) in which the small Li^+^ cations can be displaced under the application of a gate bias. On such substrates, we fabricated the same set of interdigitated electrodes as the one used for silica gating (see a schematic of the device in Figure 2a and Figure S2). The benefit of such gates is best revealed by impedance measurements, shown in Figure 2b. At high frequency, the capacitance is low. In this regime, ions cannot move, and only the dielectric behavior of the substrate is observed. As a result of the substrate being thick (several hundred µm), the resulting capacitance is even weaker than in thin silica films. At low frequency, on the other hand, ions can move to form an ionic interfacial layer, and the obtained capacitance becomes high (up to 2 µF·cm^−2^). The transition frequency between these two regimes depends on the temperature and reflects the thermally activated transport of Li^+^ ions within the substrate. In practice, ion displacement limited the gate sweep rate range, and the strongest modulations were obtained with low bias sweep rates (1 mV·s^−1^ typically, see Figure 2e). Note that the capacitance value obtained is similar to the one obtained with a liquid or ion gel electrolyte, while the device can be air-operated without handling any liquid.

As bias was applied over the lithium-ion glass substrate, current modulation was observed in the HgTe NC film (see Figure 2c and Figures S3 and S4). Current modulation was observed for temperatures down to 130 K (see Figure 2d); below this value, the ions froze. This enables the operation of the FET from room temperature down to this value, which is a broader range than the typical one of liquid electrolyte (acetonitrile often used as solvent freezes at −45 °C) or ionic glass [31,32,33] (operating range 180–300 K). However, despite the higher capacitance than with silica, the on/off ratio is not improved due to higher leakage inherent to the gating method based on ion displacement. Table 1 summarizes some figures of merit relative to various gate technologies for a HgTe NC-based field effect transistor.

A striking feature is the observation of p-type only conduction (Figure 2c), whereas the material was ambipolar when coupled to silica gate. This means that the lithium-ion glass substrate behaves as a carrier selective charge injector. This behavior likely results from an asymmetry in the mobility of Li^+^ cations and their vacancies in the lithium-ion glass substrate. We have estimated the hole mobility at 250 K to be 6 × 10^−3^ cm^2^·V^−1^·s^−1^. This is typically three times lower than the value measured for SiO_2_ gates; this reflects a carrier density dependence of the mobility.

**Figure 3:**
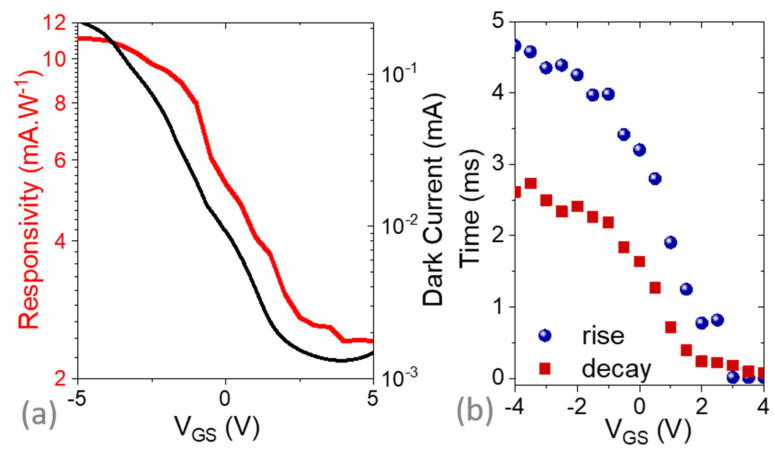
(**a**) Dark current (black line) and responsivity (red line) for the device depicted in Figure 2a as a function of the applied gate bias. (**b**) Rise and decay time (defined as the time for the signal to change from 10% to 90% of its final value) as a function of applied gate bias (see Figure S5 for a time trace). Illumination was ensured by a 1.55 µm laser diode at 110 mW.cm^−2^; spectra were averaged 500 times The drain-source bias was set to 500 mV. Measurements were conducted at 250 K.

As the purpose of this device is its use as a phototransistor, we tested its potential under illumination at 1.55 µm. As expected for such NC films, the material was photoresponsive and we observed a responsivity around R = 5 mA·W^−1^ (floating gate), which can be tuned by applying a gate bias, as shown in Figure 3a. Though higher responsivity (up to R = 11 mA·W^−1^) can be obtained under negative gate bias, the strongest photocurrent modulation is obtained under positive gate bias since this operating condition makes the material more intrinsic. As often observed in NC-based phototransistors, the change in the photocurrent magnitude comes with a change in the response dynamics [27] (see Figure 3b). Faster responses are obtained when majority carriers are removed (i.e., positive gate bias here). These changes in dynamics reflect the change in trap filling when the gate bias is applied [46]. With Li gating, the response time spans from 500 µs to 5 ms depending on the applied gating bias. The former value is itself a decade longer than the value obtained with the silica gate, which is also associated with a much weaker photoresponse. In other words, the FET configuration enables tuning of the device photoresponse while leaving the gain bandwidth product almost unaffected.

### 3.3. Introduction of a Photonic Structure to Enhance the Absorption

As with most NC-based light sensors in this configuration, the device faces a limitation. Due to hopping conduction, the mobility and the diffusion length are limited. As a result, the effective light absorption only occurs over a distance much shorter than the light absorption depth (>µm for HgTe NC in the SWIR [47]). Electromagnetic simulations revealed that the film absorbs around 10% of the incident light at the NC band edge. To overcome this limitation, we updated the design of the device to couple the NCs to a photonics structure [48], while maintaining the same interdigitated geometry (Figure S6). The initial device relied on interdigitated electrodes with 10 µm wide digits and 10 µm spacing between digits. We then tuned the geometrical factors (film thickness, period and size of the digits) in order to maximize the absorption in magnitude and linewidth. Compared to the previous work by Gréboval et al. [25], also dealing with a phototransistor coupled to a light resonator, the change in substrate from a single crystal of SrTiO_3_ to a Li-based ceramic which strongly scatters light (Figures S4 and S5) raised the need for an additional design.

**Figure 4:**
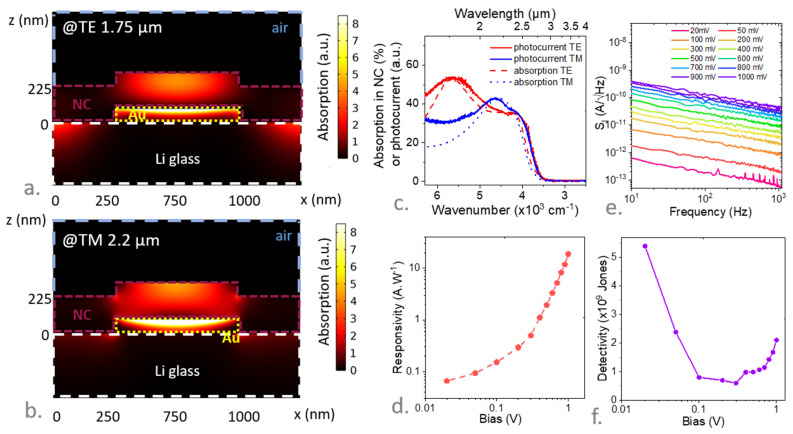
(**a**) (resp (**b**)) Simulated absorption map at the resonance of the TE @1.75 µm (resp TM at 2.2 µm) polarization. (**c**) Simulated absorption (dashed lines) and experimental photocurrent (solid lines) spectra within the NC film (i.e., excluding losses in contact) for TE and TM polarization. (**d**) Responsivity measured at 180 K for a signal at 1 kHz as a function of applied bias for the device with light resonators under broadband radiation resulting from a blackbody at 980 °C. (**e**) Noise current spectral density as a function of signal frequency measured under various applied biases. (**f**) Specific detectivity measured at 180 K (for a signal at 1 kHz) as a function of applied bias for the device with light resonators.

We first matched a resonance in the TM polarization (magnetic field parallel to the electrode ribbons) with the band-edge energy. This first resonance, at 2.2 µm, is a vertical Fabry-Perot resonance highlighted by its lack of dispersion (see Figure S7b). The energy of this resonance is driven by the film thickness (Figure S8b) but is very weakly affected by the digit width (Figure S9). The obtained mode is spatially located on top of the gold pad (Figure 4c) as the cavity is formed between the gold and the air interface. By doing so, we minimized the loss in the metal, which is estimated to be around 5.5% of the total absorption. Details about the electromagnetic design can be found in the supporting information.

Furthermore, by tuning the period of the grating we can induce a second resonance in the TE polarization (electric field parallel to the electrodes ribbons). We chose the period of the grating and digit size to slightly blueshift this resonance with respect to the band edge (around 1.75 µm). By doing so, we broadened the global response of the device. This mode is a guided mode resonance [49,50,51] (GMR), whose energy is driven by the period of the grating. This mode is dispersive, as revealed by the dispersion map (see Figure S7a), and spatially located at the top of the gold electrode as revealed by the simulated absorption map (Figure 4a). The obtained absorption within the NCs (i.e., leading to the generation of the photocurrent) is expected to reach 40% for non-polarized light (see Figure 4c).

Since the period of the grating was set at 1.5 µm for a 750 nm wide digit, we used e-beam lithography for its fabrication (see Figure S10). The obtained photoresponse spectrum was now strongly polarized (see Figure 4c), with a TE mode clearly blue shifted with respect to the TM mode. Compared to the simulation, the photocurrent presented a good match both in energy and linewidth as long as we accounted for the scattering of the Li substrate. It is worth noting that the photocurrent spectrum was also significantly red shifted from the absorption shown in Figure 1a. This shift was the combination of two effects: (i) first, we conducted a ligand exchange procedure that tends to increase inter-NC coupling and lead to a redshift. In addition (ii), the photocurrent was measured at low temperature (180 K), while the HgTe NC band gap redshifts upon cooling [47,52].

The responsivity of the device was now much higher and reached 10 A·W^−1^ (Figure 4d and Figure S14) under moderate electric field: this value is three orders of magnitude larger than the one reported for the same film deposited on conventional interdigitated electrodes without resonances. As has already been pointed out by Chu et al. [51], the enhanced responsivity splits into two contributions: absorption enhancement is responsible here for a factor of 4 in the performance increase; the remaining factor of 250 is due to photogain [53] (i.e., recirculation of one of the carriers while the other one stays trapped). Indeed, the external quantum efficiency of the device is around 500% (R = 10 A·W^−1^ for 2.5 µm cut-off wavelength and under broadband blackbody radiation).

In a nanocrystal array, charge transport occurs through hopping. Under illumination, electron-hole pairs get generated and one carrier can easily get trapped while the second one is transported to the electrode. To ensure sample neutrality, the electrode will reinject this carrier, which can recirculate several times until the trapped charge gets recombined [54]. The gain magnitude is the ratio of the trapped carrier lifetime to the transit time. The latter is directly connected to the device size. Thus, a smaller device favors gain [55].

The factor of 250 for the gain value is further subdivided into a factor of 10 that arises from an increase in the electric field (i.e., a 10 times narrowing of the electrode spacing) and a factor of 25 that we ascribe to a more efficient hopping transport as the device size approaches the diffusion length [51]. Noise in this device is limited by a 1/f contribution [56,57] (see the inset of Figure 4e), which currently limits the detectivity around D* ≈ 5 × 10^9^ Jones and corresponds to a noise equivalent power (NEP) at around 1 pW·Hz^−1/2^ for a signal at 1 kHz. This is, nevertheless, a factor of 5 (D* = 10^9^ jones) above the value obtained for the same material (same band gap and same surface chemistry) without a resonator compared to that achieved for silica [51].

## 4. Conclusions

To summarize, we have demonstrated that a lithium-ion glass substrate can be used as an all-solid back gate for a NC-based FET. This gate leads to a capacitance as high as 2 µF.cm^−2^, similar to a liquid electrolyte while considerably easing the device’s manipulation and its future integration. Moreover, the gating remains effective over a broad range of temperatures from room temperature down to 130 K, matching the targeted operating temperature range for short- and mid-wave infrared sensors. Interestingly, the gating favors hole injection compared to silica gating. In a second step, we demonstrated a light management strategy for a phototransistor based on this lithium-ion glass. We demonstrated the combination of two light resonators, one based on a guided-mode resonance and the second one relying on a Fabry-Pérot mode. This combination of two resonances enables broad band enhancement in light absorption and responsivity as high as 10 A.W^−1^; this value is a thousand times larger than the one obtained for interdigitated electrodes without any light management strategy. This enhancement can be split over a factor of 4 for absorption and a factor of 250 for photogating.

## Figures and Tables

**Table 1:** Typical ranges of use for solid state gating of HgTe nanocrystal-based field effect transistors.

Gate Technology	Dielectric	Ionic Glass	Ion Gel Electrolyte	Solid State Li Electrolyte
**Temperature range**	4 K–300 K	180 K–260 K	300 K	150–300 K
**Sweep-rate range**	Fast (several V·s^−1^)	Intermediate (0.1 V·s^−1^)	Slow (1 mV·s^−1^)	Better below 10 mV·s^−1^
**Subthreshold slope**	3400 mV/decade	1200 mV/decade	152 mV/decade	1200 mV/decade
**Gate voltage range**	<60 V (dielectric breakdown)	Up to 10 V at 200 K	<3 V (electrochemical stability of the electrolyte)	Tested up to 7 V

## Data Availability

The data used to support the findings of this study are included within the article and its supplementary information.

## Supplementary Materials

The following supporting information can be downloaded at: https://www.mdpi.com/article/10.3390/ma16062335/s1, Figure S1: thickness determination of HgTe film using profilometer. Figure S2: schematic of the field effect transistor. Figure S3: Transfer curve of the Li-gated FET at various temperatures. Figure S4: schematic of the responsivity setup. Figure S5: Photoresponse under illumination. Figure S6: refractive index of the Li-based ceramic. Figure S7: extinction coefficient spectra of the HgTe NC film. Figure S8: Schematic of device with resonator. Figure S9: Dispersion map in TE and TM polarizations. Figure S10: Absorption spectra for various film thicknesses. Figure S11: Absorption spectra for various gold digit sizes. Figure S12: Microscopy images of the resonator. Figure S13: Microscopy images of the resonator functionalized by NC film. Figure S14: IV curve of the resonator device. Figure S15: Responsivity of the resonator device as a function of frequency and power.
