# Supplementary material for: Lithium-Ion Glass Gating of HgTe Nanocrystal Film with Designed Light-Matter Coupling
Source: Materials (Basel). 2023 Mar 14;16(6):2335. doi: 10.3390/ma16062335 (PMC10054404; doi:10.3390/ma16062335)
Supplement: Supplementary file 1 [file materials-16-02335-s001.zip › materials-2206114-supplementary.pdf]

Supporting information for

Lithium-ion glass gating of HgTe nanocrystal film with designed light-matter coupling

Stefano Pierini[1$], Claire Abadie[2$], Tung Huu Dang[1], Adrien Khalili[1], Huichen Zhang[1], Mariarosa Cavallo[1], Yoann Prado[1], Bruno Gallas[1], Sandrine Ithurria[3], Sébastien Sauvage[4], Jean Francois Dayen[5,6], Gregory Vincent[2], Emmanuel Lhuillier[1*]

[1] Sorbonne Université, CNRS, Institut des NanoSciences de Paris, INSP, F-75005 Paris, France.
[2] ONERA - The French Aerospace Lab, 6, chemin de la Vauve aux Granges, BP 80100, 91123 Palaiseau, France.
[3] Laboratoire de Physique et d'Etude des Matériaux, ESPCI-Paris, PSL Research University, Sorbonne Université Univ Paris 06, CNRS UMR 8213, 10 rue Vauquelin 75005 Paris, France.
[4]Université Paris-Saclay, CNRS, Centre de Nanosciences et de Nanotechnologies, 91120, Palaiseau, France.
[5] Université de Strasbourg, IPCMS-CNRS UMR 7504, 23 Rue du Loess, 67034 Strasbourg, France
[6]Institut Universitaire de France, 1 rue Descartes, 75231 Paris cedex 05, France

$ authors have equal contributions

*To whom correspondence should be sent: el@insp.upmc.fr

Table of content

1. Material growth and characterization....2
2. Field effect transistor fabrication....3
3. Electromagnetic design....5
   3.1. Determination of optical index....5
   3.2. Design of light resonators....5
   3.3. Device fabrication....8
   3.4. Device performances....9
4. REFERENCES....10

1. Material growth and characterization

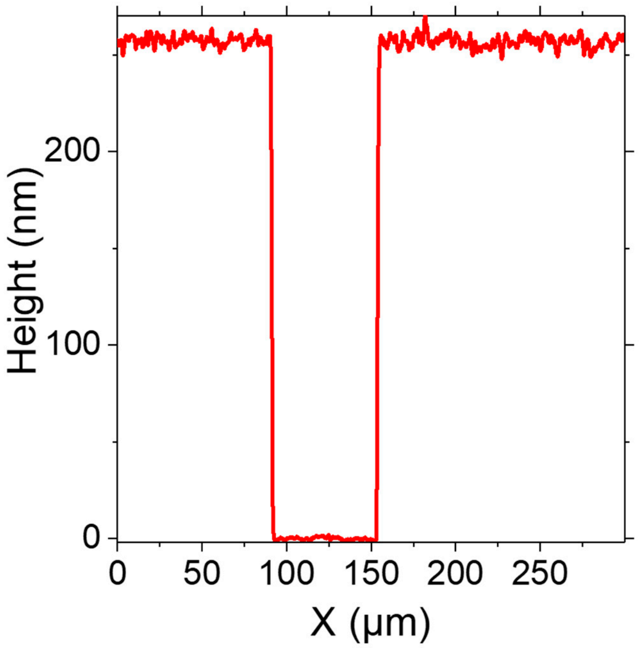

Figure S1 HgTe NC film height profile along a groove.

2. Field effect transistor fabrication

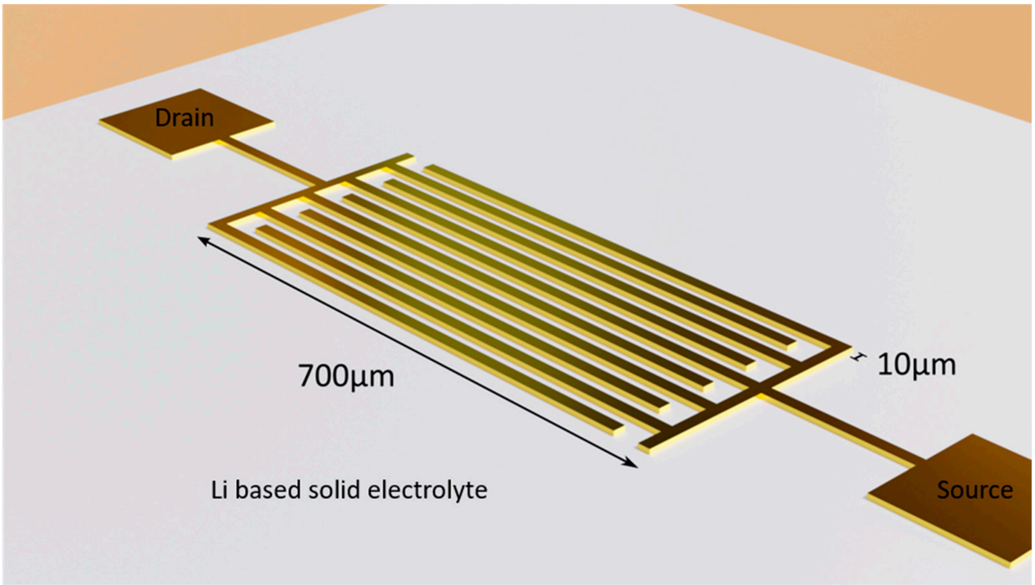

Figure S2 Schematic of the interdigitated electrodes used in the first part of the paper.

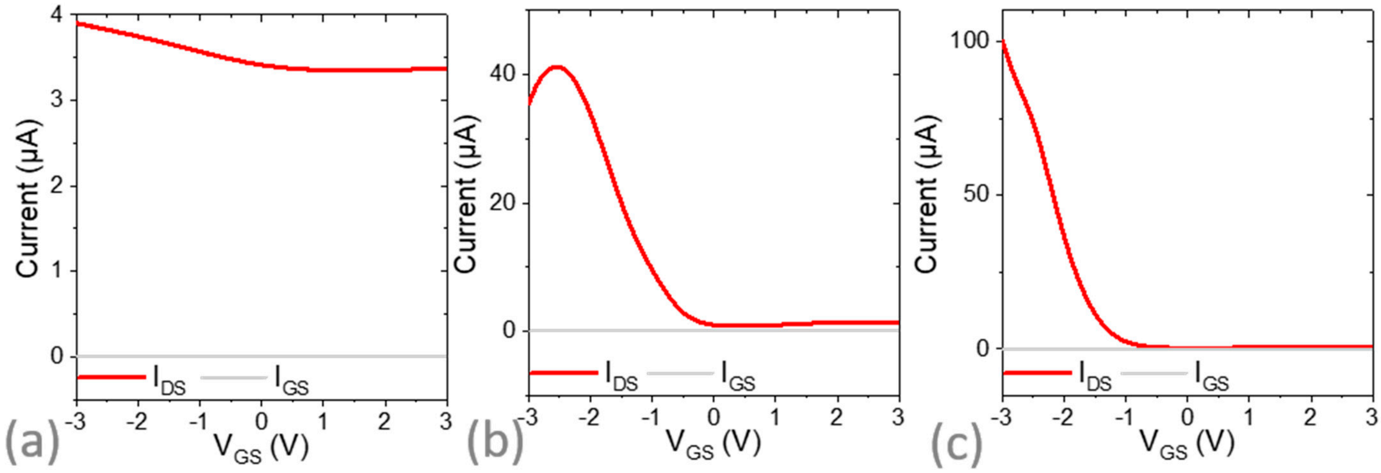

Figure S3 Transfer curves for HgTe NC film deposited on Li-based substrate measured at 130 K (a), 150 K (b) and 200 K (c). In all cases the drain source bias is set at 0.5 V.

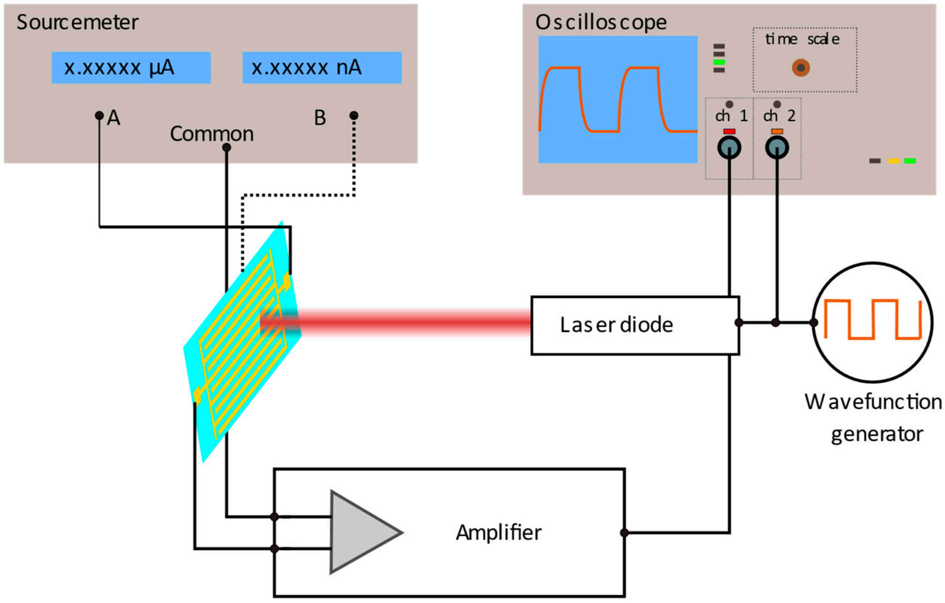

Figure S4 Scheme of connections for time response and responsivity measurements

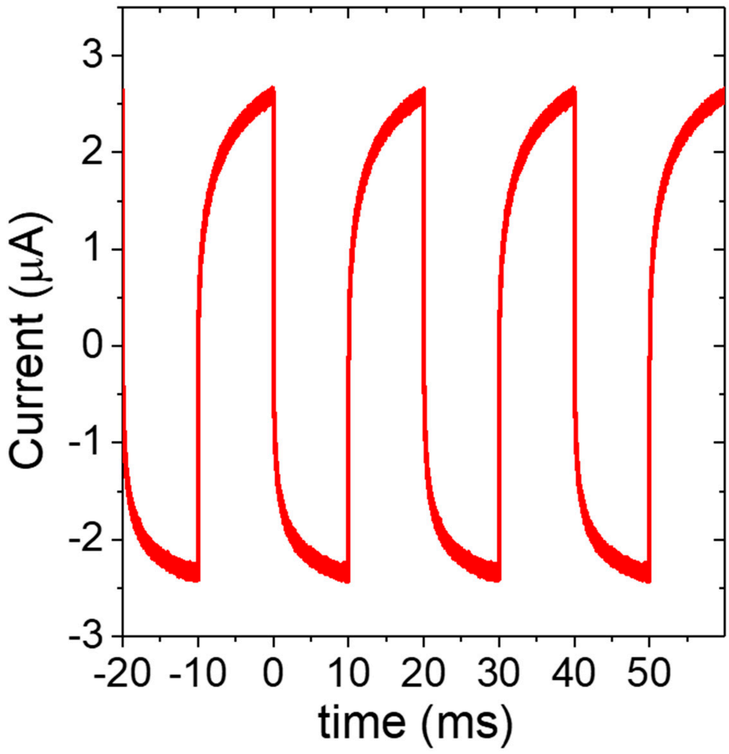

Figure S5 Time response of the Li based phototransistor. Data have been acquired in AC mode to suppress the average value.

3. Electromagnetic design

3.1. Determination of optical index

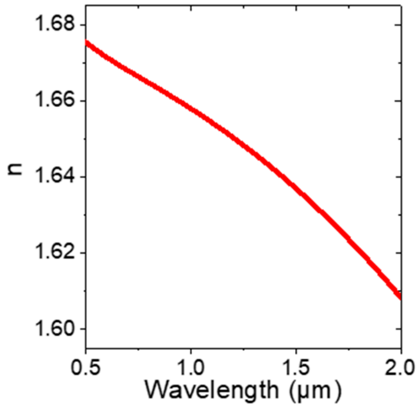

Figure S6 Optical index of the Li based substrate.

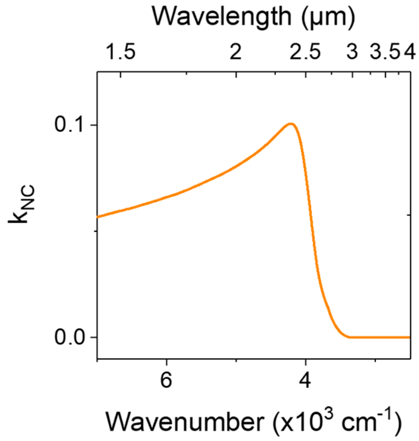

Figure S7 Extinction coefficient spectrum of the HgTe NC film.

3.2. Design of light resonators

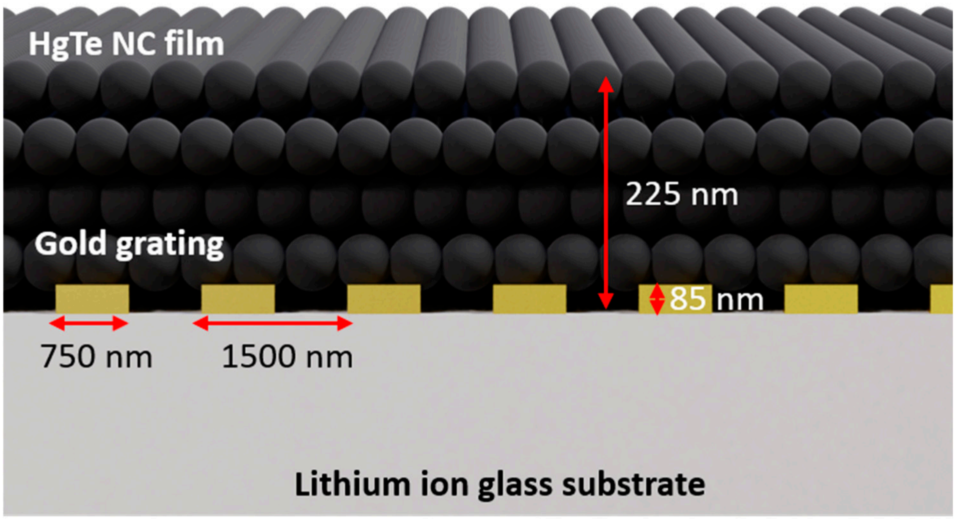

Figure S8 Side view of the device coupling Li based substrate and light resonators.

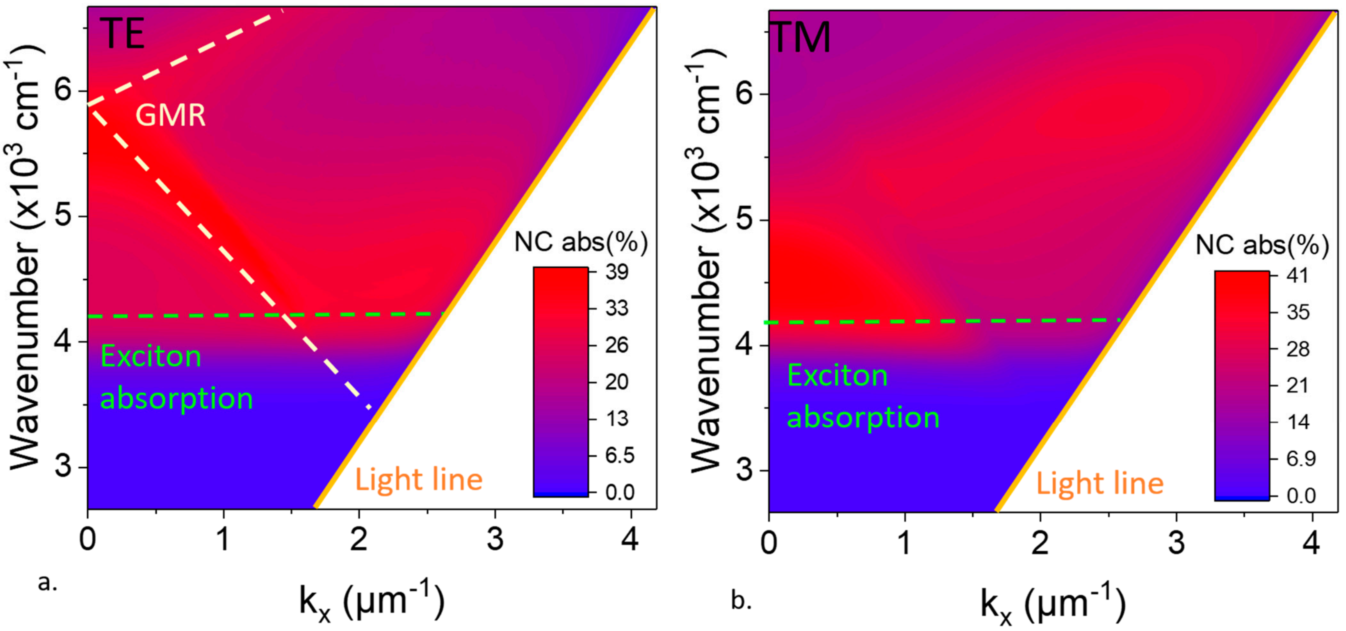

Figure S9 Dispersion map (absorption, within the NCs only, as a function of in plane wave vector and incoming wavenumber) for TE (a) and TM (b) polarization

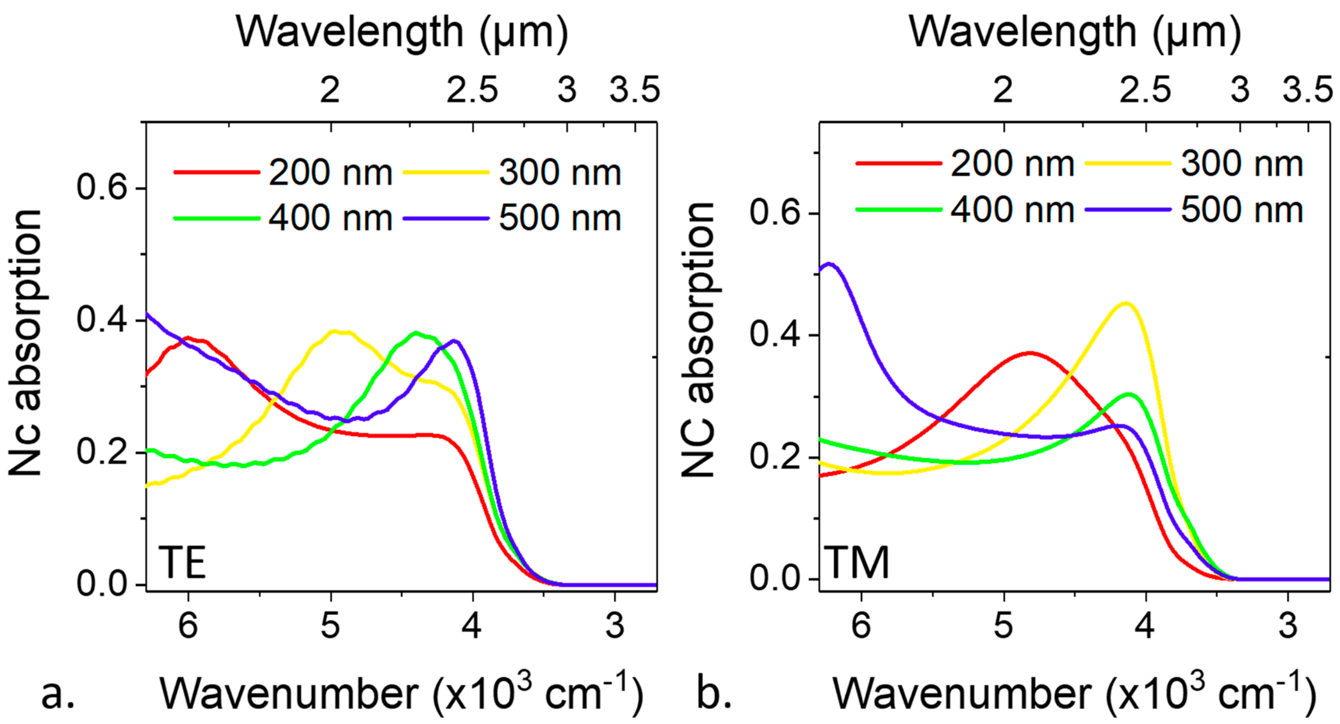

Figure S10 Absorption spectra of the device, within the NCs only, for various film thicknesses along TE (a) and TM (b) polarizations.

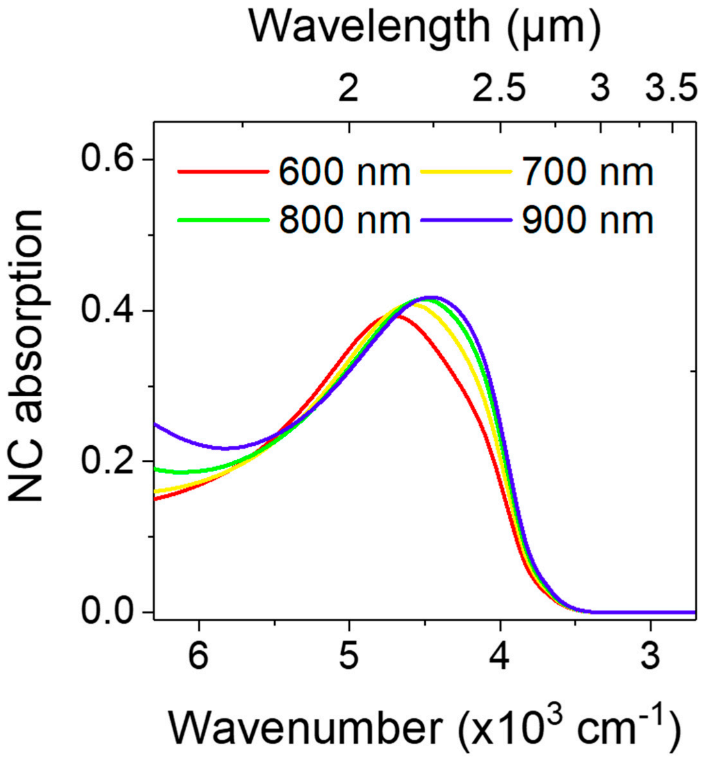

Figure S11 Absorption spectra of the device, within the NCs only, for various sizes of the digit along the TM polarization.

3.3. Device fabrication

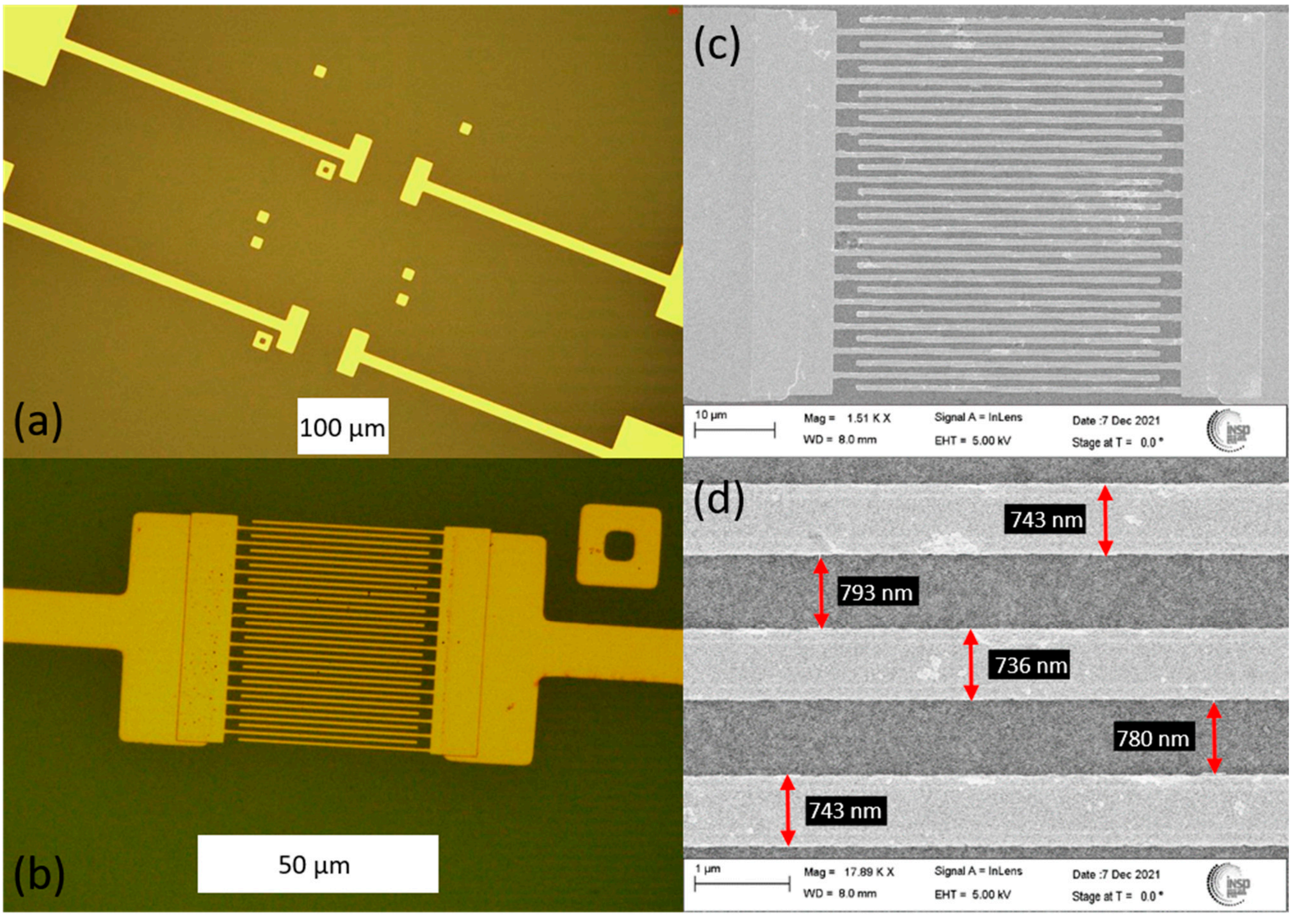

Figure S12a. Optical microscopy image of the contact pads obtained at the end of the optical lithography step. b. Optical microscopy image of the interdigitated pattern after the e-beam lithography step. c. Scanning electron microscopy image of the interdigitated pattern after the e-beam lithography step. d. Zoom on part c.

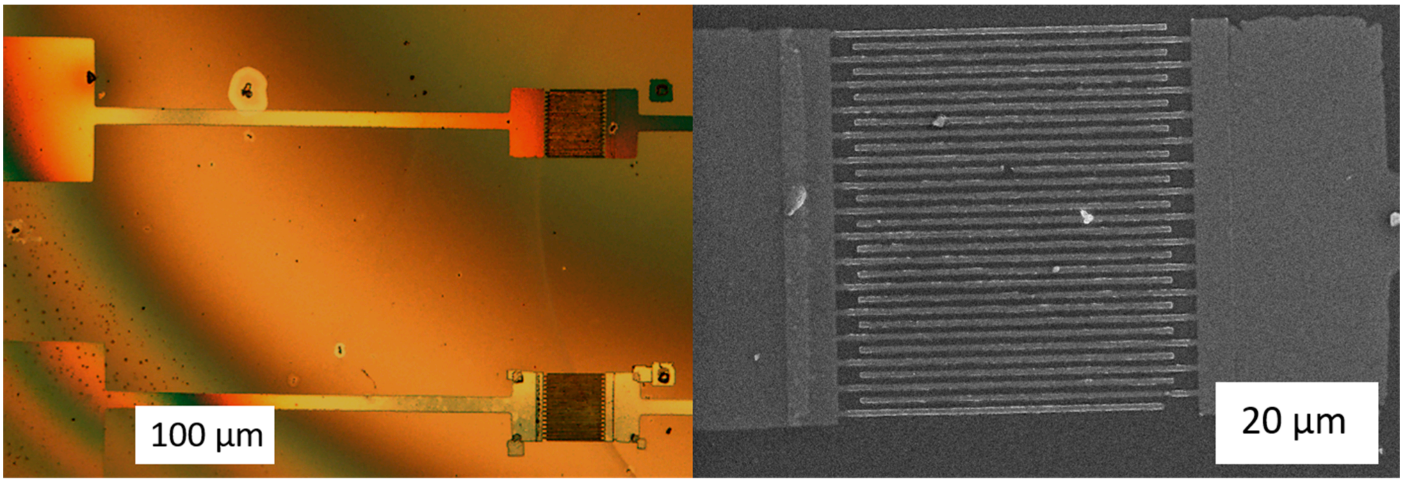

Figure S13 Image of the electrodes after NC film functionalization. a. Optical image and (b.) scanning electron microscopy image.

3.4. Device performances

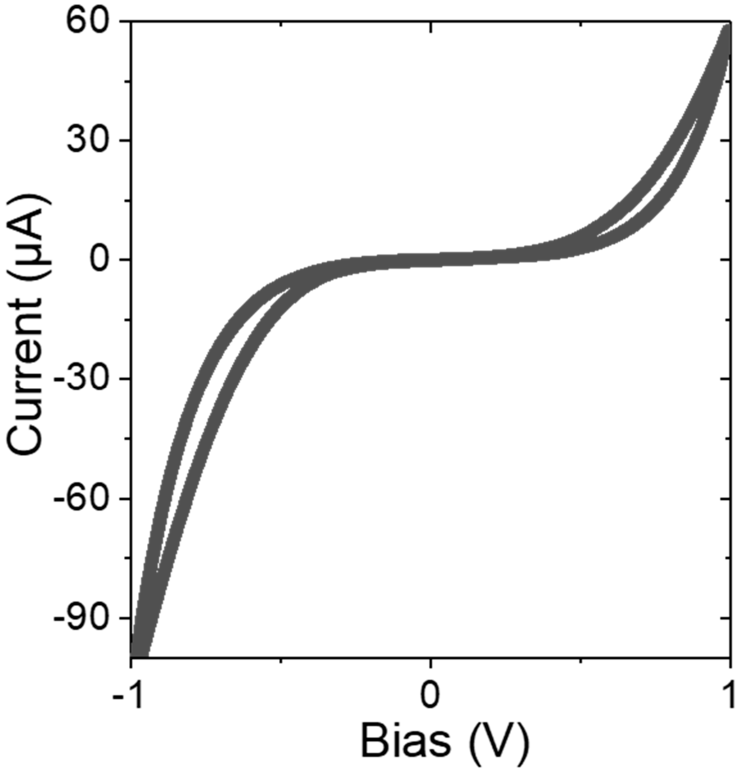

Figure S14 IV curve of the device at 300 K.

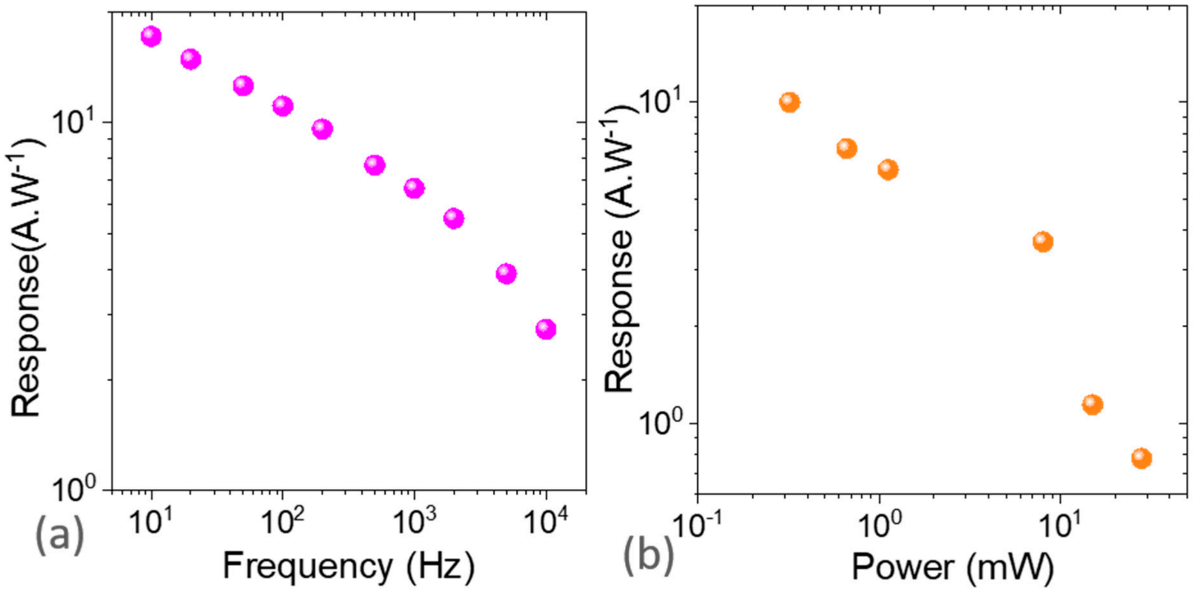

Figure S15 Reponsivity as a function of frequency (a) and optical power delivered by the illuminating laser diode (b). The laser diode operates at 1.55 µm. Drain source bias is set to 700 mV for these two graphs. Measurements are conducted at 180 K.

4. REFERENCES

[1] Olmon R L, Slovick B, Johnson T W, Shelton D, Oh S-H, Boreman G D and Raschke M B 2012 Optical dielectric function of gold *Phys. Rev. B* **86** 235147

[2] Rastogi P, Chu A, Dang T H, Prado Y, Gréboval C, Qu J, Dabard C, Khalili A, Dandeu E, Fix B, Xu X Z, Ithurria S, Vincent G, Gallas B and Lhuillier E 2021 Complex Optical Index of HgTe Nanocrystal Infrared Thin Films and Its Use for Short Wave Infrared Photodiode Design *Adv. Opt. Mater.* **9** 2002066
